# Osteopontin Level in Synovial Fluid Is Associated with the Severity of Joint Pain and Cartilage Degradation after Anterior Cruciate Ligament Rupture

**DOI:** 10.1371/journal.pone.0049014

**Published:** 2012-11-15

**Authors:** Mika Yamaga, Kunikazu Tsuji, Kazumasa Miyatake, Jun Yamada, Kahaer Abula, Young-Jin Ju, Ichiro Sekiya, Takeshi Muneta

**Affiliations:** 1 Department of Joint Surgery and Sports Medicine, Tokyo Medical and Dental University, Tokyo, Japan; 2 International Research Center for Molecular Science in Tooth and Bone Diseases (Global Center of Excellence Program), Tokyo Medical and Dental University, Tokyo, Japan; 3 Department of Cartilage Regeneration, Tokyo Medical and Dental University, Tokyo, Japan; University of Michigan, United States of America

## Abstract

**Objective:**

To explore the molecular function of Osteopontin (OPN) in the pathogenesis of human OA, we compared the expression levels of OPN in synovial fluid with clinical parameters such as arthroscopic observation of cartilage damage and joint pain after joint injury.

**Methods:**

Synovial fluid was obtained from patients who underwent anterior cruciate ligament (ACL) reconstruction surgery from 2009 through 2011 in our university hospital. The amounts of intact OPN (OPN Full) and it’s N-terminal fragment (OPN N-half) in synovial fluid from each patient were quantified by ELISA and compared with clinical parameters such as severity of articular cartilage damage (TMDU cartilage score) and severity of joint pain (Visual Analogue Scale and Lysholm score).

**Results:**

Within a month after ACL rupture, both OPN Full and N-half levels in patient synovial fluid were positively correlated with the severity of joint pain. In contrast, patients with ACL injuries greater than one month ago felt less pain if they had higher amounts of OPN N-half in synovial fluid. OPN Full levels were positively correlated with articular cartilage damage in lateral tibial plateau.

**Conclusion:**

Our data suggest that OPN Full and N-half have distinct functions in articular cartilage homeostasis and in human joint pain.

## Introduction

Osteoarthritis (OA) is a group of diseases and mechanical abnormalities involving degradation of articular cartilage and subchondral bone. Clinical manifestations of OA may include joint pain, tenderness, stiffness, creaking, locking of joints, and local inflammation [1]. It was reported that OA affects 27 million people in the U.S. in 2005 and it is estimated that 80% of the U.S. population will have radiographic evidence of OA by age 65 [1]. These statistics strongly indicate that both prevention of cartilage loss and promotion of cartilage repair in the recovery of joint function are important issues to address [2].

Currently, the major therapeutic strategy for OA is based on conservative treatments, such as muscle exercise with medications, to relieve joint inflammation and pain [3]. However, these treatments are not always satisfactory because they are not powerful enough to inhibit OA progression nor can they promote cartilage repair. To overcome these problems and to develop a new radical treatment for OA, many efforts have been concentrated to understand the molecular pathogenesis of OA. One approach to understand the molecular pathogenesis of OA may be the identification and characterization of the genes involved in joint development and homeostasis. Studies have identified gene sets with altered expression levels in the joint during the progression of OA and RA. These genes include MMP-13 [4]–[7], OPN [8], [9], ECRG4 [10], hYKL40 [11], and hYKL39 [12]. In this study, we focused on analyzing the molecular function of OPN in the pathogenesis of human OA.

OPN is an O-glycosylated phosphoprotein produced by a variety of tissues and cells including osteoblasts, chondrocytes, synoviocytes, and T cells [13]. It was identified as a major non-collagenous bone matrix protein as well as an inflammatory cytokine [13]–[16]. Previous studies reported that OPN is susceptible to proteolytic fragmentation extracellularly to form different sized protein fragments [17]–[20]. Full length OPN (OPN Full) is shown to increase in OA synovial fluid and articular cartilage while its N-terminal fragment, OPN N-half, a proteolytic fragment produced by thrombin, is increased in the proinflammatory situation such as rheumatoid arthritis (RA) [8], [9], [21], [22].

**Figure 1:**
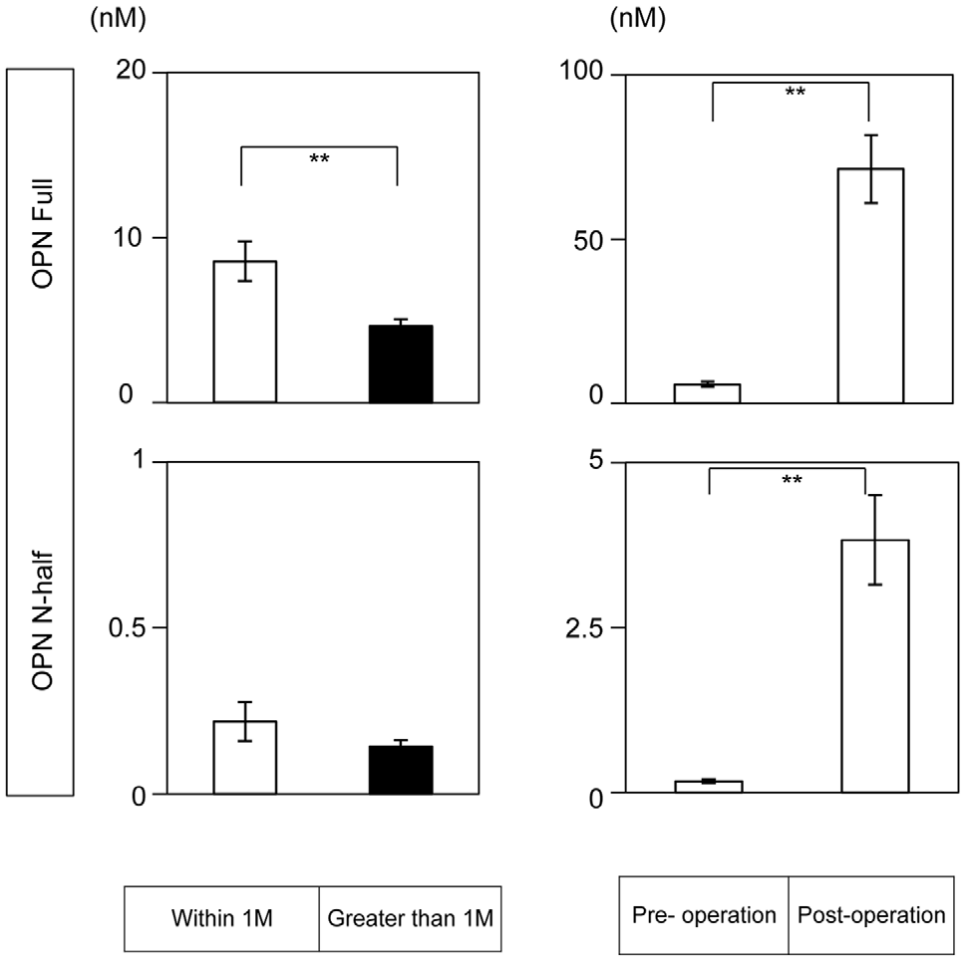
Kinetics of OPN Full and N-half in synovial fluid after joint injury. (Left panels) Time course changes of OPN Full (upper panel) and OPN N-half (lower panel) protein levels in synovial fluid after ACL rupture. Open bar: within 1 month after rupture n = 14, Closed bar: greater than 1 month after rupture n = 68. (Right panels) OPN Full (upper panel) and OPN N-half (lower panel) protein levels in synovial fluid collected from pre (n = 23) and post (n = 93) ACL reconstruction surgery. Data are indicated mean+/− SEM. **; p<0.01.

Since OPN contains cryptic binding sequences for several different receptors, fragmented OPN proteins are considered to have different functions in distinct pathological conditions [23]–[25]. It is shown that OPN Full interacts with integrin alpha-v βeta-3 through the GRGDS motif and activates various molecules involved in MAPK and NFkB signaling pathways [23], [26]. It also binds to CD44, and is considered to be involved in the process of inflammation, immune response, and bone metabolism [13], [27]. C-terminal sequence of OPN N-half contains SVVYGLR motif, which is reported as a cryptic alpha-1 or alpha-4 integrin binding sequence that is exposed by thrombin in an inflammatory situation. The level of OPN N-half is reported to significantly increase in the synovial fluid from rheumatoid arthritis (RA) patients in comparison with that of OA patients [21], [22]. Despite of these previous studies, physiological roles of OPN Full and OPN N-half in joint maintenance have not studied in detail.

Studies utilizing OPN knockout mice have reported that aging-associated and instability-induced OA were accelerated in the absence of OPN [28]. In contrast, Yumoto et al reported that inflammation-induced articular cartilage degradation was significantly inhibited in the absence of OPN [29]. These data strongly suggest that OPN has complex roles in joint homeostasis and in the pathogenesis of arthritis by modulating multiple targets of cells in the joint. However, these studies have not elucidated distinct roles of OPN Full and N-half as both proteins were eliminated in these mice.

Since OPN is considered as a proinflammatory cytokine, we hypothesized that OPN levels in the synovial fluid were correlated with joint inflammation and pain. In this study, to examine our hypothesis and to further explore the distinct roles of OPN Full and N-half in joint homeostasis, we analyzed OPN Full and N-half levels in synovial fluid after joint injury and performed correlation analyses with various clinical parameters. Here we report that OPN Full and OPN N-half levels are associated with the severity of joint pain and articular cartilage damage in humans. We consider the two most important subjects in OA research to be the elucidation of the mechanism of joint pain and the mechanism of articular cartilage degradation in humans, both of which are quite difficult to answer using animal models.

**Figure 2:**
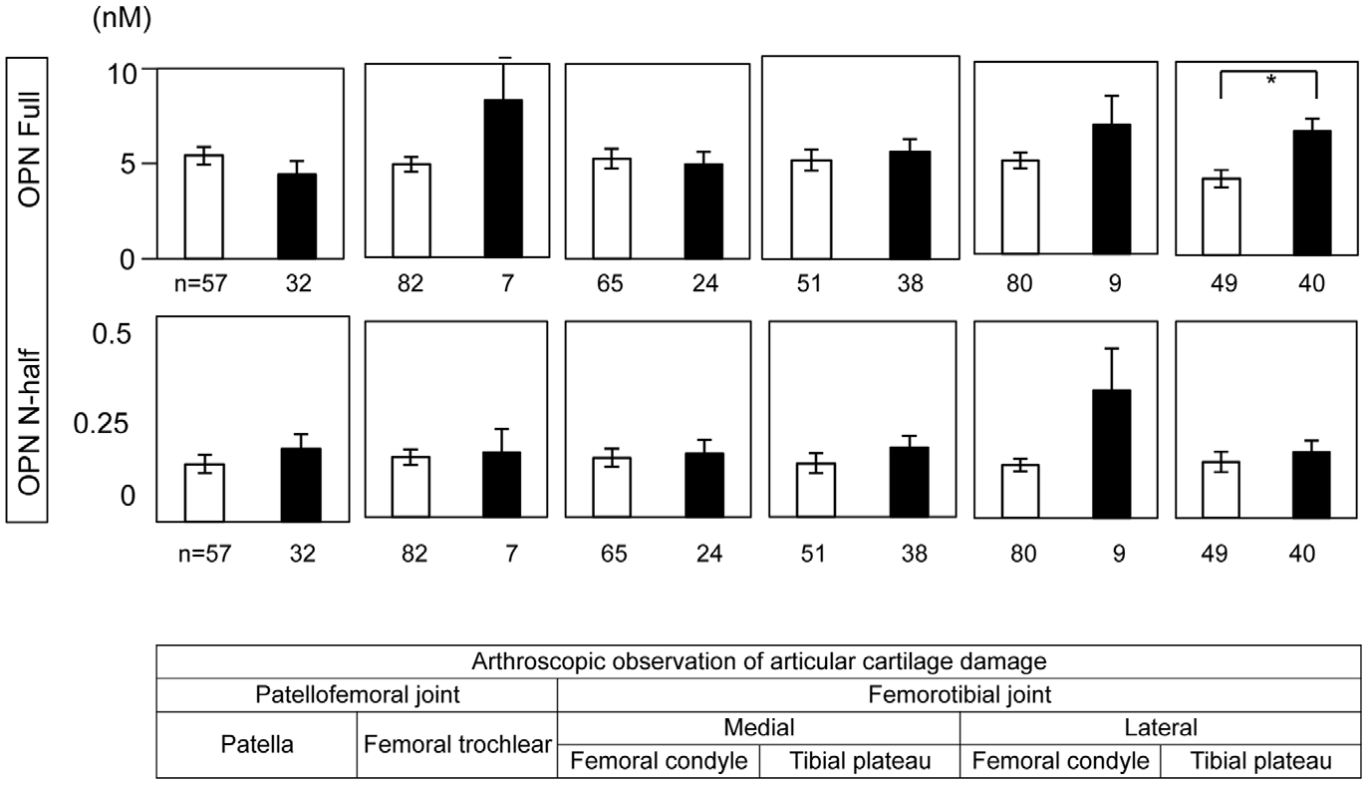
OPN Full levels in synovial fluid are positively correlated with joint damage in lateral tibial plateau. Severity of articular cartilage damage was scored according to the protocol described by Asano et al [32] with minor modification (Table S1). Open bar; intact articular cartilage (score = 1) Closed bar; damaged articular cartilage (score = 2∼6). Number of samples is indicated below each column. Data are indicated mean+/− SEM. *; p<0.05.

## Methods

### Human Tissue Samples

This study was approved by the Ethics Committee of Tokyo Medical and Dental University. All patients included in this study gave their full written, informed consent for participation prior to the operative procedure. For minors/children, we obtained informed written consent from their parents or guardians. In this study, we regarded ACL reconstruction patients as a high risk OA group as it was reported that more than 30% of ACL reconstruction patients develop radiographic OA on an average of 7.8 years after surgery, with most patients having no evidence of OA at the time of rupture [30], [31]. One hundred and twenty-two (Male: 81, Female: 41) patients aged 14–48 (average 25) years (M: 15–47, F: 14–48), who underwent ACL reconstruction surgery in our university hospital from January 2009 through December 2011, were enrolled in the study. Duration of ACL injury until reconstruction surgery of patients was 2 weeks to 20 years (average 12.9 months). Exclusion criteria included the history of severe meniscus tear. Synovial fluid was aspirated from the knee joint just before ACL reconstruction surgery and 4 days post-surgery. All surgical procedures were performed by expert joint surgeons in our department. Aspirated synovial fluid was centrifuged to remove debris and stored immediately at −80°C.

**Figure 3:**
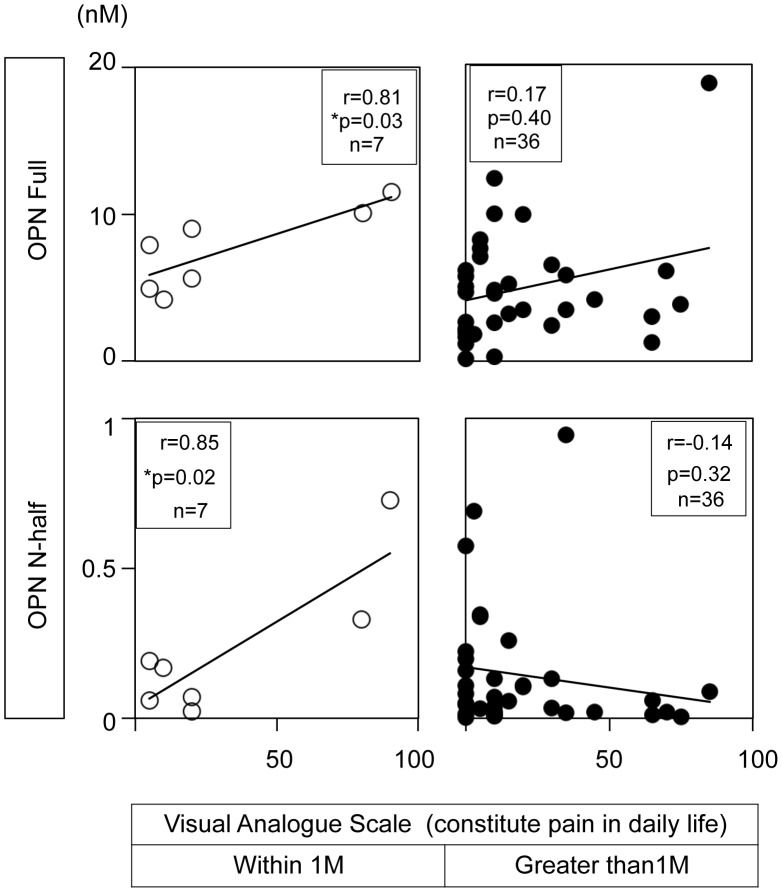
OPN Full and N-half levels in synovial fluid are positively correlated with the severity of joint pain within a month after ACL rupture. VAS indicating constitutive pain in a daily life (see Table S2) was collected in the examination room before consultation. Open circle; VAS from patients within 1 month after ACL rupture. Closed circle; VAS from patients who ruptured ACL greater than 1 month ago. Number of samples, correlation coefficient, and p value are indicated in each figure.

**Figure 4:**
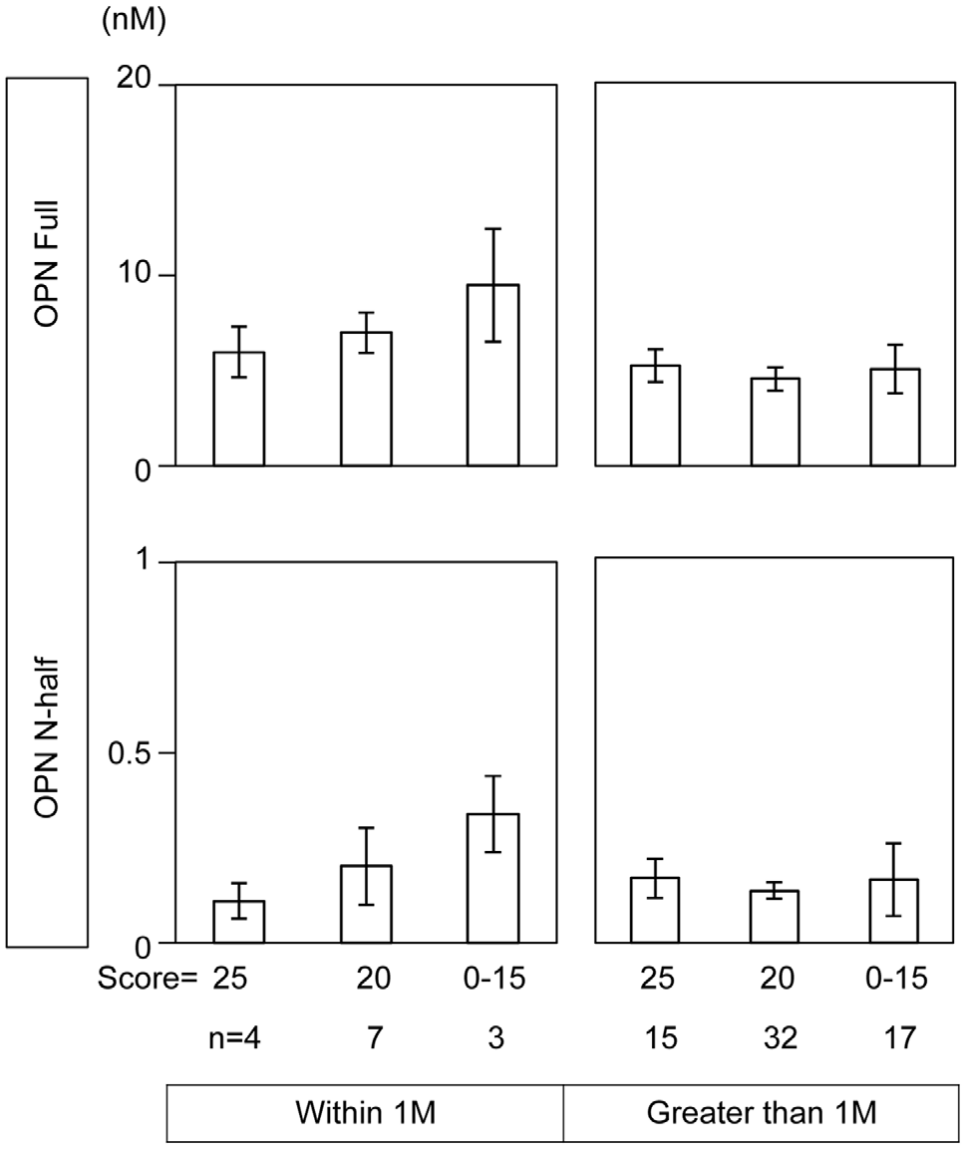
Correlation of OPN Full and N-half levels in synovial fluid with Lysholm scores. Lysholm scores (see Table S3) were collected in an examination room by expert joint surgeons in our university hospital. The basis of the classification of Lysholm score was 25:feel no pain at any time, 20:feel not so severe or tolerable pain even after severe exertion, 0–15:feel severe or intolerable pain sometimes in a daily life. Numbers of samples are indicated below each figure.

**Figure 5:**
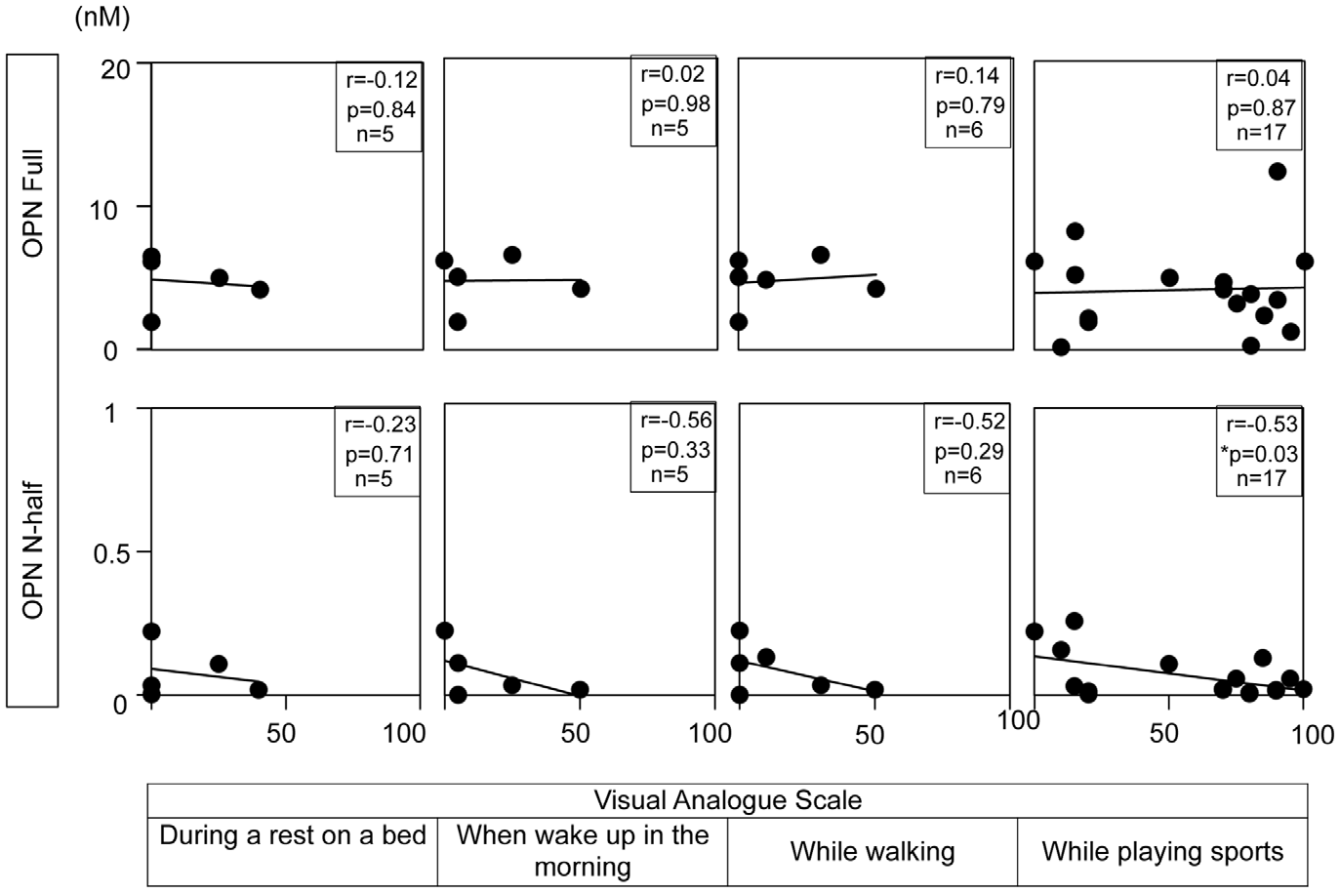
OPN N-half levels in synovial fluid are negatively correlated with the severity of joint pain in patients who ruptured ACL greater than 1 month ago. VAS was collected from patients who ruptured ACL more than 1 month ago using a questionnaire described in Table S2 in an examination room at our university hospital. Number of samples, correlation coefficient, and p value are indicated in each figure.

### Quantification of OPN Full and N-half in Synovial Fluid

Since OPN is shown to process by thrombin in an inflammatory environment [21], [22], we quantified both intact OPN protein (OPN Full) and the thrombin-processed form (OPN N-half) in this study. Protein levels of OPN Full and N-half in synovial fluid were quantified independently using an ELISA kit according to the manufacturer’s protocol (IBL Co. Ltd. Tokyo Japan). In some experiments, we divided the subjects into 2 groups, within 1 month and greater than 1 month, because our preliminary data indicated that the volume of synovial fluid quickly increased after the joint injury and usually returned to the basal level after 1 month (data not shown).

**Figure 6:**
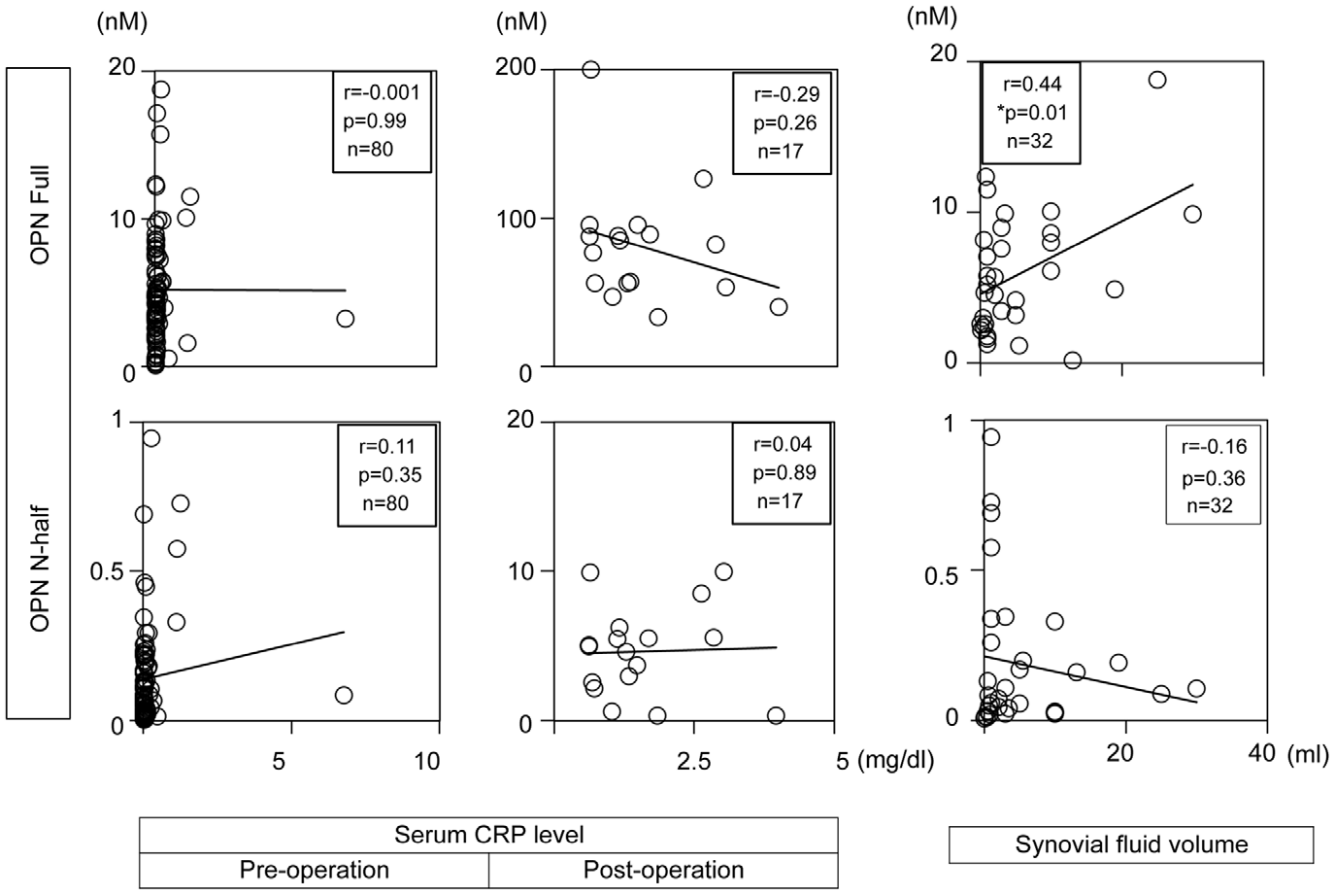
OPN Full and N-half levels in synovial fluid are independent from systemic inflammatory status but associate with joint inflammation. (Left and Middle panels) Blood samples were collected pre- and post-operation (at day 4) and serum CRP levels were quantified at the clinical laboratory of our university hospital. (Right panels) Synovial fluid was collected just before operation in a surgery room. Number of samples, correlation coefficient, and p value are indicated in each figure.

### Arthroscopic Observation of Articular Cartilage Damage (TMDU Score)

The severity of articular cartilage damage was scored during ACL reconstruction surgery according to the classification protocol described by Asano et al with modification [32]. Details are shown in Table S1. These scores were determined mainly by the two skilled operators during the surgery by mutual consent.

### Visual Analogue Scale (VAS) and Lysholm Score

VAS was collected in the examination room before consultation (one day before ACL reconstruction surgery). Lysholm scores were collected by expert joint surgeons in our university hospital during consultation. Details are shown in Table S2 and S3.

### Statistical Analysis

All the analyses were performed by a double-blind method. Mann-Whitney’s U-test or Kruskal–Wallis test followed by Steel–Dwass multiple comparison tests were employed to analyze the differences between groups. Pearson’s correlation coefficient test was employed for correlation analyses. Values of *P*<0.05 were considered significant.

## Results

### Kinetics of OPN Levels in Synovial Fluid after Joint Injury

As shown in Fig. 1, OPN Full levels were significantly decreased with time after ACL rupture. In contrast, OPN N-half levels remained low and did not alter significantly between the two time periods after ACL rupture (left panels). Since it is reported that OPN gene expression is upregulated in response to injury and inflammation in various organs including bone [33], [34], we analyzed OPN protein levels at pre- and 4 days post-operation. Both OPN Full and N-half levels surged by almost 10-fold at 4 days post ACL reconstruction (right panels). The base line level of OPN Full was almost 10-fold of that of OPN N-half.

### Synovial Fluid OPN Levels are Positively Correlated with the Severity of Articular Cartilage Damage in Lateral Tibial Plateau

Matsui et al reported that the development of aging-associated and instability-induced osteoarthritis was accelerated in OPN deficient mice [28]. To examine if synovial fluid OPN levels were correlated with the severity of articular cartilage damage after ACL rupture in humans, we compared OPN Full and N-half levels in synovial fluid with the arthroscopic observation of cartilage damage in patellofemoral and femorotibial joints. As shown in Fig. 2, we found that synovial fluid OPN Full levels were positively correlated with the damage of articular cartilage in lateral tibial plateau in the femorotibial joint. In contrast, we did not observe any correlation between OPN N-half levels and articular cartilage damage.

### Synovial Fluid OPN Levels and the Severity of Joint Pain

Fig. 3 shows the correlation of synovial fluid OPN levels with constitutive pain in daily life. As shown in this figure, both OPN full and N-half levels were positively correlated with the severity of constitutive joint pain in patients suffering from ACL rupture within 1 month. These correlations were statistically significant (left panels). In contrast, we did not observe any correlation in the patients suffering from ACL rupture greater than 1 month post-injury (right panels). Interestingly, we observed a negative relationship between OPN N-half levels and VAS at this stage. These results seemed to be reproducible since we observe similar tendency between OPN levels in synovial fluid and Lysholm scores (Fig. 4). To further analyze the correlation between the severity of joint pain and synovial fluid OPN levels in patients following the acute inflammation stage, we evaluated the correlation between daily activities and joint pain in patients whose ACL injury had surpassed one month. We compared VAS of patients when they rest on a bed (first column), wake up (second column), walk (third column), and play sports (forth column) with OPN levels (Fig. 5). In parallel with the results in Fig. 3 right panels, we did not observe any correlation between OPN Full levels and VAS (Fig. 5 upper panels) at this stage. Interestingly, OPN N-half levels tended to negatively correlate with VAS at this stage, which was statistically significant when they played sports (p = 0.03, Fig. 5 lower 4th column).

Since local inflammation is considered to be a major contributor for joint pain, we next examined if synovial fluid OPN levels are correlated with the severity of systemic and local inflammation. As shown in Fig. 6, we did not observe any correlation between OPN levels in synovial fluid and serum C-reactive peptide (CRP) levels, a marker for systemic inflammation (Fig. 6 left and middle panels). However, we observed significant positive correlation between OPN Full levels and the volume of synovial fluid (Fig. 6 right upper panel). Since synovial fluid volume usually increases during joint inflammation, our results suggest that OPN Full levels may associate with joint inflammation [35]. In contrast, OPN N-half levels seemed to negatively correlate with joint inflammation (Fig. 6 right lower panel).

## Discussion

OPN is a phosphorylated acidic glycoprotein with diverse functions including cell adhesion, chemoattraction, immunomodulation, and cell differentiation [13], [17]. OPN is considered to be involved in the pathogenesis of human OA since its expression level is enhanced with OA progression [22]. Previous in vitro and knockout mice experiments indicated that OPN is influential in articular cartilage metabolism in both physiological and pathological conditions [28], [29], however the role of OPN in the pathogenesis of human OA was still unclear. To explore the pathophysiological roles of OPN in human OA, we compared OPN expression levels in synovial fluid with various clinical conditions such as arthroscopic observation of articular cartilage and joint pain. In this manuscript we report that OPN levels were correlated with the severity of articular cartilage damage in lateral tibial plateau and joint pain. This is the first report showing that OPN is involved in joint pain in human OA.

In the present study we demonstrated that kinetics of OPN Full and N-half levels were different in synovial fluid. Both OPN Full and N-half levels surged almost 10-fold by 4 days after ACL reconstruction surgery. This result is comparable with previous reports indicating that OPN is an early response gene against various stress signals [36], [37]. We observed that levels of OPN full in synovial fluid were significantly higher within 1 month than greater than 1 month after ACL rupture. In contrast, OPN N-half levels remained low and did not alter significantly between the two time periods after ACL rupture (Fig. 1 left panels). Since we could not detect OPN mRNA expression in the synovial membranes obtained from patients with ruptured ACL by RT-PCR analyses (data not shown), we expect that OPN gene expression is immediately upregulated at the point of joint injury and quickly shut down during the healing process. Posttranslational OPN processing by thrombin to produce OPN N-half may occur and quench very quickly in synovial fluid after joint injury.

With regard to the correlation of OPN levels and articular cartilage damage, we found that OPN Full levels are positively correlated with the severity of articular cartilage damage in lateral tibial plateau (Fig. 2). Lateral tibial plateau is the region where bone bruise is most frequently observed by X-ray and MRI (magnetic resonance imaging) after ACL injury [38]. This suggests that OPN Full may accelerate inflammation-induced cartilage degradation. Fig. 6 indirectly supports this idea that OPN Full levels are positively correlated with the amount of synovial fluid, which is usually positively correlated with the severity of joint inflammation [35]. OPN knockout mice experiments also support this idea since OPN knockout mice were resistant in inflammation-induced articular cartilage degradation [29]. In contrast, we did not observe any correlation between OPN N-half levels and articular cartilage damage. One reason for that may be due to the kinetics of OPN N-half in synovial fluid after joint injury. We showed that OPN N-half levels quickly quenched after joint damage while cartilage damage usually progresses by the month. The other possible reason for that may be due to the differences of receptor usage between OPN Full and N-half. Further studies are required to elucidate the functional differences between OPN Full and N-half on cartilage metabolism.

The most interesting finding of this study was the correlation of OPN levels with the severity of joint pain. We observed that OPN Full and N-half levels are positively correlated with the severity of joint pain in patients who suffered from ACL rupture within 1 month. Since OPN functions as a proinflammatory cytokine and regulates PTGS2 and iNOS expression [9], [27], we speculate that OPN may mediate the expression of pain inducers, such as prostaglandin E2 and nitric oxide, at the acute inflammatory stage after ACL rupture. In contrast, we observed a negative correlation between OPN N-half levels and joint pain in patients whose ACL rupture surpassed one month, when the acute inflammation has already quenched. Although we do not know yet the functional differences between OPN Full and N-half, these data suggest that OPN N-half may have an inhibitory function against OPN Full. To evaluate this hypothesis, we examined the effect of OPN N-half on the regulation of PTGS2 mRNA that was induced by OPN Full in chondrogenic ATDC5 cells. However, our preliminary data indicated a subtle effect of OPN N-half on PTGS2 expression (data not shown). Since OPN is also reported as an intrinsic inhibitor of inflammation in cartilage [9], further molecular analyses of OPN Full and N-half are necessary to elucidate the specific roles of these proteins in joint pain.

In summary, we found that OPN levels were correlated with the severity of articular cartilage damage in lateral tibial plateau and joint pain. These results suggest that OPN may be an important target to relieve OA patients from severe joint pain and cartilage degradation.

## Supporting Information

Table S1 Classification of arthroscopic observation of articular cartilage damage. Severity of articular cartilage damage was scored according to the protocol described by Asano et al [32] with minor modification as shown in the table.
(DOCX)

Table S2 Visual Analogue Scale (VAS) for pain. VAS was collected using a questionnaire described in the table.
(DOCX)

Table S3 Lysholm score for pain. Lysholm scores were collected in an examination room by expert joint surgeons in our university hospital. Classification of each score was described in the table.
(DOCX)
