# Supplementary material for: Osteopontin Level in Synovial Fluid Is Associated with the Severity of Joint Pain and Cartilage Degradation after Anterior Cruciate Ligament Rupture
Source: PLoS One. 2012 Nov 15;7(11):e49014. doi: 10.1371/journal.pone.0049014 (PMC3499533; doi:10.1371/journal.pone.0049014)
Supplement: Table S1 — Classification of arthroscopic observation of articular cartilage damage. Severity of articular cartilage damage was scored according to the protocol described by Asano et al [32] with minor modification as shown in the table. (DOCX) [file pone.0049014.s001.docx]

Table S1 Classification of arthroscopic observation of articular cartilage damage

A: Score

| 1 | Normal |
| --- | --- |
| 2 | Softening |
| 3 | Fissure |
| 4 | Fibrillation or Ulcer-less than half cartilage thickness |
| 5 | Fibrillation or Ulcer-more than half cartilage thickness |
| 6 | Cartilage defect-Full thickness |
| 7 | Bone defect |

B: Region

| Patellofemoral joint | Patella | (1, 2, 3, 4, 5, 6,7) |
| --- | --- | --- |
|  | Femoral trochlear | (1, 2, 3, 4, 5, 6,7) |
| Femorotibial joint | Medial femoral condyle | (1, 2, 3, 4, 5, 6,7) |
|  | Medial tibial plateau | (1, 2, 3, 4, 5, 6,7) |
|  | Lateral femoral condyle | (1, 2, 3, 4, 5, 6,7) |
|  | Lateral tibial plateau | (1, 2, 3, 4, 5, 6,7) |
