# Supplementary material for: Osteopontin Level in Synovial Fluid Is Associated with the Severity of Joint Pain and Cartilage Degradation after Anterior Cruciate Ligament Rupture
Source: PLoS One. 2012 Nov 15;7(11):e49014. doi: 10.1371/journal.pone.0049014 (PMC3499533; doi:10.1371/journal.pone.0049014)
Supplement: Table S2 — Visual Analogue Scale (VAS) for pain. VAS was collected using a questionnaire described in the table. (DOCX) [file pone.0049014.s002.docx]

Table S2 Visual Analogue Scale (VAS) for pain

| Constitutive pain in daily life | 0  Mild  100  Severe |
| --- | --- |
| During a rest on a bed | 0  Mild  100  Severe |
| When woke up in the morning | 0  Mild  100  Severe |
| While walking | 0  Mild  100  Severe |
| While playing sports | 0  Mild  100  Severe |
