# Supplementary material for: Osteopontin Level in Synovial Fluid Is Associated with the Severity of Joint Pain and Cartilage Degradation after Anterior Cruciate Ligament Rupture
Source: PLoS One. 2012 Nov 15;7(11):e49014. doi: 10.1371/journal.pone.0049014 (PMC3499533; doi:10.1371/journal.pone.0049014)
Supplement: Table S3 — Lysholm score for pain. Lysholm scores were collected in an examination room by expert joint surgeons in our university hospital. Classification of each score was described in the table. (DOCX) [file pone.0049014.s003.docx]

Table S3 Lysholm score for pain

| 25 | None |
| --- | --- |
| 20 | Inconsistent and slight during severe exertion |
| 15 | Marked during severe exertion |
| 10 | Marked on or after walking more than 2 km |
| 5 | Marked on or after less than 2 km |
| 0 | Constant |
